# Functional Genomics for Plant Breeding 3.0

**DOI:** 10.3390/ijms25042131

**Published:** 2024-02-09

**Authors:** Fatemeh Maghuly, José Manuel Cruz-Rubio

**Affiliations:** Plant Functional Genomics, Institute of Molecular Biotechnology (IMBT), Department of Biotechnology (DBT), University of Natural Resources and Life Sciences (BOKU), Muthgasse 18, 1190 Vienna, Austria; jose.cruz-rubio@boku.ac.at

Functional genomics, as a scientific discipline, has significantly transformed the landscape of plant breeding in recent years. With the rapid advancements in omics technologies, particularly genomics, researchers have gained an in-depth understanding of the molecular mechanisms governing plant traits and their responses to diverse environmental conditions. The integration of functional genomics into plant breeding has enabled breeders to make informed a priori decisions accelerating the development of improved crop varieties with enhanced characteristics, such as disease resistance, abiotic stress tolerance, and nutritional quality.

The intersection of functional genomics and plant breeding has not only facilitated the identification of key genes and regulatory elements but has also enhanced the selection of superior plant materials, contributing to increased crop yields, sustainability, and resilience in the face of climate change. Moreover, functional genomics has played a critical role in decoding complex traits that were previously challenging to comprehend. The in-sights derived from this research directly impact food security, agricultural sustainability, and global health.

We are delighted to present the third installment of our Special Issue series on “Functional Genomics for Plant Breeding”, which showcases a collection of research papers highlighting the latest advancements in functional genomics and their practical applications in plant breeding. This issue stands as a testament to the ongoing progress in the field and the significant contributions of researchers worldwide.

In this Special Issue, we offer a diverse set of studies exploring the intricacies of functional genomics and their relevance to enhancing plant breeding programs. These papers underscore the innovative research aimed at developing resilient, high-yielding, and sustainable plant varieties; a brief overview of these research papers is as follows.

Wu et al. [1] investigate the complex effects of FT (Flowering locus T) gene overexpression in tobacco plants, shedding light on its multifaceted impact on leaf development. The results reveal the intricate influence of FT overexpression on various biological processes, providing valuable insights into the function of FT and its homologous genes. This work underscores the importance of understanding the regulatory role of FT genes in plant growth and development, a crucial aspect of plant breeding programs.

Chen et al. [2] focus on the vital role of chloroplasts in plant photosynthesis, studying a rice green leaf mutant (*crs2*) to uncover the molecular basis of its chlorophyll deficiencies. The mutation’s impact on chloroplast protein abnormalities and photosynthetic performance is thoroughly examined, offering insights into the physiological mechanisms affecting photosynthesis and high-lighting the significance of functional genomics in enhancing photosynthetic efficiency in plant breeding.

Rodriguez-Alcocer et al. [3] report on an EMS-induced mutation in Arabidopsis thaliana leading to albinism and lethality at the seedling stage. By identifying the underlying genetic mutation and its effects on the splicing of *At2g04030* transcripts, this study provides insights into the deregulation of genes encoding plastid-localized proteins, advancing our understanding of chloroplast-localized proteins. This research extends the understanding of how genetic variations impact plant development, an essential consideration in plant breeding.

Yang et al. [4] identify a major QTL (*qSCM4*) associated with lodging resistance in rice, a significant concern in modern plant breeding. Through genetic analysis and DNA sequencing, they pinpoint the candidate gene responsible for this trait, offering valuable insights for enhancing rice cultivars and understanding the genetic factors contributing to plant strength and resilience.

The study by Salava et al. [5] focuses on the NGA gene family, specifically the NGATHA transcription factor, and its role in various plant species. This comprehensive analysis provides a detailed understanding of the gene family’s structure, function, and evolution, laying the foundation for further exploration of its contributions to plant developmental processes.

These published papers collectively underscore the vital role of functional genomics in advancing plant breeding. They highlight the importance of in-depth genetic and molecular research in the development of resilient and productive plant varieties, ultimately contributing to global food security and sustainability.

We would like to extend our appreciation to all the authors who have contributed their valuable research to this Special Issue, as well as the reviewers and editorial team for their dedicated efforts in ensuring the quality and integrity of the published papers. The collective knowledge shared in this Special Issue will undoubtedly benefit the scientific community and propel advancements in the field of plant breeding.

Thank you for your attention and dedication to the “Functional Genomics for Plant Breeding 3.0” Special Issue. We encourage all readers to explore these insightful papers and harness the knowledge they offer to continue improving plant breeding practices and addressing the challenges of our time.
